# Aerosol emission from playing wind instruments and related COVID-19 infection risk during music performance

**DOI:** 10.1038/s41598-022-12529-2

**Published:** 2022-05-21

**Authors:** Carl Firle, Anke Steinmetz, Oliver Stier, Dirk Stengel, Axel Ekkernkamp

**Affiliations:** 1 GP Practice, Dr. Claudia-Isabella Wildfeuer, 10715 Berlin, Germany; 2 Physical and Rehabilitation Medicine, Department of Trauma, Reconstructive Surgery and Rehabilitation Medicine, University Medicine Greifswald, Greifswald, Germany; 3 Siemens AG, Technology, 13623 Berlin, Germany; 4 BG Kliniken-Klinikverbund Der Gesetzlichen Unfallversicherung gGmbH, Berlin, Germany; 5 Department of Trauma, Reconstructive Surgery, and Rehabilitation Medicine, University Medicine Greifswald, Greifswald, Germany; 6 BG Klinikum Unfallkrankenhaus Berlin gGmbH, Berlin, Germany

**Keywords:** Respiration, SARS-CoV-2, Viral infection, Respiratory tract diseases, Physics

## Abstract

The pandemic of COVID-19 led to restrictions in all kinds of music activities. Airborne transmission of SARS-CoV-2 requires risk assessment of wind instrument playing in various situations. Previous studies focused on short-range transmission, whereas long-range transmission risk has not been assessed. The latter requires knowledge of aerosol emission rates from wind instrument playing. We measured aerosol concentrations in a hermetically closed chamber of 20 m^3^ in an operating theatre as resulting from 20 min standardized wind instrument playing (19 flute, 11 oboe, 1 clarinet, 1 trumpet players). We calculated aerosol emission rates showing uniform distribution for both instrument groups. Aerosol emission from wind instrument playing ranged from 11 ± 288 particles/second (P/s) up to 2535 ± 195 P/s, expectation value ± uncertainty standard deviation. The analysis of aerosol particle size distributions shows that 70–80% of emitted particles had a size of 0.25–0.8 µm and thus are alveolar. Masking the bell with a surgical mask did not reduce aerosol emission. Aerosol emission rates were higher from wind instrument playing than from speaking or breathing. Differences between instrumental groups could not be found but high interindividual variance, as expressed by uniform distribution of aerosol emission rates. Our findings indicate that aerosol emission depends on physiological factors and playing techniques rather than on the type of instrument, in contrast to some previous studies. Based on our results, we present transmission risk calculations for long-range transmission of COVID-19 for three typical woodwind playing situations.

## Introduction

The pandemic of COVID-19 has forced many countries to shut down major parts of their public life, including the music business. Virus-laden aerosols exhaled by infectious persons are a potential source of secondary SARS-CoV-2 infections^1–4^. Resulting attack rates depend on a plethora of factors, the most significant of which are the aerosol emission rate of the primary case and the dilution factor^5^. Singing indoors has early been found hazardous^5–7^ and a correlation between voice activity level and aerosol emission rate is widely accepted^8–10^. Since even silent breathing generates aerosols^11^ it is necessary to examine to which extent wind instrument playing possibly increases particle emission over normal breathing, without vocalization. Since increasingly infectious virus variants have developed during the pandemic, this question remains relevant for the music scene.

Orchestras, ensembles, conservatories, and music schools have a demand for reliable data on aerosol emission from wind instrument playing^12^. Protection from aerosol inhalation requires wearing FFP2 or N95 masks which is not feasible for the player of a wind instrument. Social distancing and face masks other than FFP2 or N95 protect susceptible persons from droplet transmission, but not from airborne transmission of SARS-CoV-2. FFP2 masks meet the same criteria as N95 masks defined by the National Institute for Occupational Safety and Health (NIOSH)^13^. FFP2 masks and N95 masks have a filtering efficiency > 95%. Protective measures against aerosol transmission need to focus on ventilation and airing. Infection risk calculations involve the aerosol emission rate as primary model parameter.

Woodwind instrument playing by an infectious person imposes disease transmission risk on other persons in the same room at two different scales of distance: short-range and long-range. Short range transmission refers to transmission occurring close to the source of aerosol, where there is still a directional respiratory jet and concentrations will be higher because little diffusion has occurred. Long range transmission refers to transmission occurring after this respiratory jet has dissipated and the aerosol is homogeneously dispersed throughout the environment.

The primary aerosol cloud in immediate vicinity of the player may carry pathogens in amounts sufficient to provoke quick infection of a susceptible person nearby. Within this nearfield of a spreader disease transmission occurs in a discontinuous manner difficult to predict^14^ and dependent on both distance and contact time. Within the short range, large droplets with limited reach convey high amounts of virus and it is a matter of coincidence whether they are inhaled or not. Susceptible persons in this region are subject to what we call short-range exposure to infection risk.

While larger droplets (aerodynamic diameters ≥ 30 µm) soon deposit due to gravitational sedimentation or shrink to aerosol particles suspensible in air^15^, fine aerosol particles (with equilibrium diameters ≤ 5 µm) stay airborne for long time and spread evenly in the surrounding air^16,17^. In absence of technical measures, such as capture-type ventilation hoods, aerosols will accumulate in the room air at largely homogeneous concentrations. When infectious, they produce a base level of infection risk which increases over time and does not depend on a susceptible person’s position in the room^1,5^. Susceptible persons are subject to what we call long-range exposure to infection risk^1,5^. Long range transmission is sensitive to the specific details of the environment. Long-range transmission probability calculation requires knowledge of aerosol particle emission rates along with exposure time, fresh air flow rate, room space volume, pulmonary ventilation rate, number of occupants, and the critical dose of infection^18,19^.

Previous studies of aerosol emission during woodwind playing gained important insight on the structure and extension of the respiratory jet and the nearfield cloud and helped to specify distancing rules for avoidance of short-range exposure. The flow structures in the vicinity of woodwind players have been investigated to characterize the shape, extensions and particle size composition of the primary exhalation cloud^20–23^. The local air concentration of aerosol has been found to decay to background level on a sub-meter length scale which indicates efficient aerosol dilution in the ambient air^22^. The plume generated by playing the clarinet has been found to be highly directional, to have high velocity, and to disperse quickly^24^. No air motion could be measured in 2 m distance in front of woodwinds which was the largest reach of aerosol clouds observed^20,21^. A reduction of aerosol emission by 50% (for particles in the size range 0.5–14 µm) to 79% (0.3–5 µm) when using a mask around the bell has been found^22,25^.

He et al. have assessed aerosol emission using aerodynamic particle sizers (APS) directly under the outlet of wind instruments to characterize the aerosol generation of different woodwind and brass instruments^26^. Their results showed that playing wind instruments, in general, generates a higher number of aerosol particles than breathing and speaking. The amount of aerosol generated was dependent on various parameters, such as instrument type, dynamics, articulation and breathing techniques^26^. This is consistent with the findings of Asadi et al. who investigated various speech components and demonstrated that aerosol production increases at loud speech or specific consonants^27^. McCarthy et al. suggest that playing wind instruments emits less aerosol than speaking and singing at high volume^28^. Their study used aerodynamic particle sizers (APS) with funnels, like in previous studies^10^. The categorization of wind instruments by aerosol emission as proposed by He et al. is not supported by McCarthy et al.^28^. They discuss that the aerosol background concentration has a major impact on aerosol measurements and recommend clean room conditions.

None of the above-named studies focusing on the nearfield allows to infer long-range transmission risk. Measurement of total particle emission rates during flute and oboe performance is complicated by the non-uniform flow of exhaled air which simultaneously emanates from different openings of the instrument and the mouth of the player. The total flow is, thus, difficult to measure whereas local particle measurements near the instrument would sample an unknown fraction of the total emission. Local aerosol concentration measurements cannot capture integral flow rates unless the total flow through a closed surface around the emission source is known.

Local collection of emitted aerosol particles using under-pressure funnels^25^ misses aerosol emanation elsewhere, without a chance to estimate the magnitude of the fugitive emission. This is less critical when measuring speaking or singing^10^ but introduces systematic errors for instruments with keyholes, particularly the flute. Therefore, McCarthy et al. used two funnels when measuring aerosol emission during flute playing^28^. Advanced collectors have been used by Stockman et al. to reduce the leakage errors during clarinet playing^24^.

The observed quick dilution of aerosols entails concentration gradients which limit the accuracy of concentration measurements by adding an accidental dependence on the sampling position. Aerosol mass concentrations, as reported by Eiche^29^, are affected by such local gradients and, additionally, by the nonstationary water content of the particles measured. Measurements of mass concentration depend on the particle size distribution and require calibration of the aerosol spectrometer for the specific kind of aerosol (solid versus liquid)^30^.

The initial spontaneous motion of emitted aerosol particles is directed upwards, due to thermal convection, but when they reach the temperature of the surrounding air they mix uniformly with the latter. Eventually, aerosol particles spread all over the premises, following global air flow. The concentrations reached determine inhalation doses of potentially infectious aerosols.

For long-range transmission risk assessment, emission rates of airborne aerosol particles have to be known, but they have not been measured during wind instrument playing, so far^19^. Measurement of the spatially uniform aerosol concentration developing after sufficient mixing inside a hermetic probe volume allows calculation of the aerosol emission rate (detailed information in Supplement S3). The entire aerosol released by the proband is diluted in the known amount of air inside the probe chamber and the total emission rate is approximately proportional to the temporal increase of the aerosol concentration. Leakage errors, as by airflow through keyholes, do not occur during such a measurement. We measure aerosol concentrations at three remote positions, away from the respiratory plume, and infer the particle emission rate.

Dependency on the setting, in particular the ceiling height, has been proposed as possible cause of discrepancy between the results of Abraham et al.^22^ and Kähler and Hain^31^. The latter study found a larger extension of the local flow zone. Low headroom accelerates the lateral spreading of aerosols and the formation of uniform concentrations in a probe volume. Accordingly, our setup uses a hermetic probe chamber with low ceiling for accelerated formation of uniform concentrations. Moreover, our probands deliver realistic music performance, rather than playing single notes^28^.

The aim of this study is to measure aerosol generation under standardized conditions during the playing of different wind instrument groups. The primary research goal is to measure the total emission rate of airborne aerosol particles while playing wind instruments, including interindividual variation. A secondary question is whether super-spreaders could be defined based on their aerosol emission rate. Lastly, ways to reduce aerosol emission were examined (outlet cover). We use the measured emission rates to predict the probability of COVID-19 transmission in three typical woodwind playing situations.

## Methods

### Study design and participants

An exploratory, cross-sectional design was used to measure aerosol emission during wind instrument playing. A convenience sample of volunteer flute, oboe, clarinet, and trumpet players was sought from professional orchestras and students at music conservatories. To qualify for the study musicians had to be at least 18 years of age and to have had professional instrumental education (university degree or similar). Exclusion criteria included acute or severe chronic respiratory diseases and symptoms of a SARS-CoV-2 infection. Recruitment of the participants was accomplished by an informing homepage and an online registration form and was conducted between the end of August and end of October 2020*.* The measurements took place during October and the first week of November 2020.

All measurements were conducted with the approval of the relevant ethics committee of the University Medical Center of Greifswald (reference number BB 131/20) in accordance with the declaration of Helsinki. Every participant gave informed written consent. The study was registered retrospectively on the German Clinical Trials Register (www.drks.de) as No. DRKS00023336.

### Experimental setup

The measurements took place in an operating theatre of the outpatient surgery centre of Unfallkrankenhaus Berlin, Germany. Room temperature and relative humidity in the theatre were constant, controlled by air conditioning. Room air was primarily filtered by carbon filters and by F7 and F9 bag filters meeting the criteria of EN 779 (average efficiency > 95% for particles of ≥ 0.4 µm).

**Figure 1:**
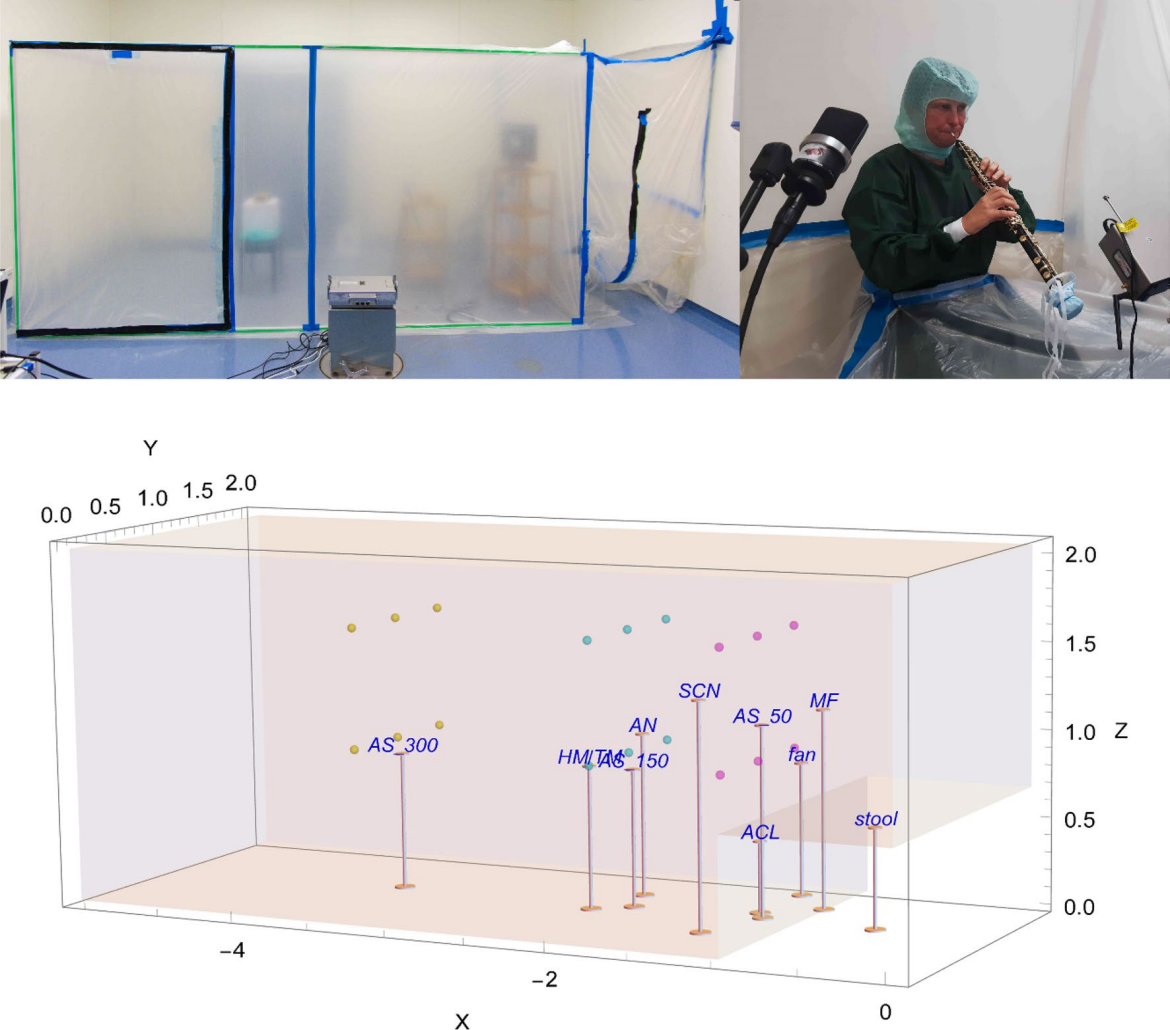
Photographs (top) and 3D-figure (bottom) of the experimental setup. Black tape on the probe chamber’s foil indicates the left and right opening slots allowing air exchange between successive measurements. Air flow measurement points are marked as coloured dots. Average room temperature was 24.0 ± 0.3 °C; average relative humidity was 42 ± 5% for flutists and 40 ± 6% for oboists. Air velocity never exceeded 0.01 m/s at the anemometer position. *AS* aerosol spectrometer, *AN* anemometer, *HM/TM* hygrometer/thermometer, *MF* microphone, *SCN* screen, *ACL* air cleaner, scales in m. The oboist gave her informed consent to publication of the photograph (right).

**Figure 2:**
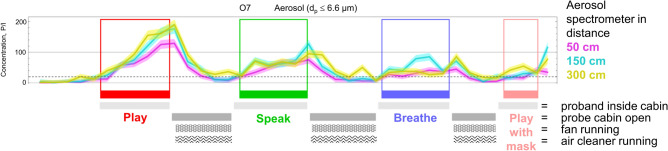
Temporal evolution of the particle air concentration during the measurement session with oboist no. 7. Solid lines indicate maximum likelihood values and shaded regions visualize the estimation uncertainty. Line colors indicate the aerosol spectrometer position and box colors indicate the task performed. The dashed line indicates the background concentration of particles inside our measurement tent as guide to the eye. Baseline values were reconditioned after each task by opening the probe chamber and flooding it with fresh air from the surrounding operating theatre. The airing was accelerated using a fan and an air cleaner (indicated by pattern bars below the boxes).

A measuring tent, the probe chamber, was set up in the operating theatre. It consisted of a 20 m^3^ cuboid constructed of aluminium bars and impermeable foil (see Fig. 1) to prevent air exchange between the inner of the cuboid and the outside during measurements. Between measurements the stale air inside the cuboid was exchanged by opening the empty probe chamber at both ends. A fan and an air purifier with HEPA filter (high-efficiency particulate air H13, CARD 210 m^3^/h) accelerated the air exchange after each task. Figure 2 shows an example of time dependent aerosol concentrations during a complete measurement session, featuring oboist no. 7. Airing between measurements went on until aerosol concentrations fell below 50 P/l on all three spectrometers for more than 4 min. The corresponding plots of all participants are available in our repository^32^ in the directory fig/Particle Counts. All participants were clothed in a surgical gown and hood and locked in after entry from the side of the probe chamber. During measurements, the upper part of the body was inside the probe chamber, tightly enfolded around the waistline by the sealing foil (see Fig. 1). We obtained written informed consent from the participant for publishing the image in an online open-access publication.

Spatially resolved measurement of air speed inside the closed cuboid with one participant sitting inside indicated < 0.01 m/s at all positions (measured by a testo 405 i anemometer, see Fig. 1). Three aerosol spectrometers (Grimm Aerosol Spectrometers 1.109 and 11-D) were placed in 50 cm (11-D), 150 cm (1.109) and 300 cm (1.109) distance from the participant. A computer monitor was placed to the participants’ left sight showing scores or text. To record the sound as audio-track a microphone was placed on the right. The thermo-hygrometer (Voltcraft DL-220THP*)* and the anemometer were placed next to the aerosol spectrometer positioned at 150 cm distance, see Fig. 1. Device specifications are reported in Supplement S1.

Aerosol concentrations were measured by the three aerosol spectrometers in the unit particles/litre, categorized by size into 31 bins ranging from > 0.25 to > 35 µm, at collection intervals of 6 s. Relative humidity (RH, unit: %) and air velocity (unit: m/s) were measured at 150 cm distance from the participant. Every 2 s air speed was recorded, and every 60 s temperature and humidity. All devices recorded continuously during a whole session, the start and end times were logged by a html-java-script.

### Tasks

Flute and oboe players were asked to play Mozart’s Oboe Concerto in C major K. 314 or Mozart’s second Flute Concerto in D major K. 314, respectively (same concerto, originally arranged for flute by Mozart himself). Clarinet and trumpet players played a piece of their own choice^32^. Each recording lasted 20 min. Subsequently, participants were asked to read a text (the beginning of Hermann Hesse’s “Der Steppenwolf”, in German) for 20 min, followed by 20 min of quiet breathing. The probands were asked to perform all tasks as normal as possible to achieve realistic breathing patterns, sound volume, and manner of speaking.

### Data evaluation

The Grimm aerosol spectrometers 1.109 and 11-D use slightly different particle size bins which we transformed into the new size bins > 29.9, > 25.1, > 12.8, > 6.6, > 3.0, > 1.6, > 0.8, > 0.4, > 0.25 µm, as described in Supplement S1. Particles with diameters up to 6.6 µm are further combined to the size bin “aerosol” whereas larger particles form the bin “droplets”. Details of the emission rate calculation are reported in Supplements S2 and S3 and in the document “data processing.pdf” in our repository^32^. All reported estimation uncertainties are calculated by the bootstrap method, as explained in Supplement S2.

The aerosol emission rates determine inhalation doses of potentially infectious aerosol particles and allow long-range transmission probability calculations, as outlined in Supplement S4.

Data evaluation and statistics were all performed with Wolfram Mathematica 13.0.1 (www.wolfram.com/mathematica). Data sets, supplementary data and figures are available in our repository^32^. For group comparisons we use the two-sided Wilcoxon–Mann–Whitney test at significance level 5%. The Kolmogorov–Smirnov test was applied to distribution fits, at significance level 5%.

## Results

Descriptive and demographic data of participants are reported in Table 1.

**Table 1:** Descriptive and demographic data of musicians.

		Flute player	Oboe player	Clarinet player	Trumpet player
n		19	11	1	1
Gender identity	female:male	16 : 3	5 : 6	0 : 1	0 : 1
Age [years]	mean	47 (15)	51 (12)	28	26
Height of participants [cm]	Female	166 (6)	168 (11)		
	Male	182 (3)	188 (9)	174	182
Weight of participants [kg]	Female	65 (12)	66 (9)		
	Male	81 (6)	84 (11)	75	90
Respiratory disease	None	18	11	1	1
	Allergy-induced bronchial asthma	1			
Nicotine consumption	None	17	7		1
	Previously	1 (3 py)	2 (1 py, 3 py)		
	Currently	1 (12 py)	2 (7 py, 35 py)	1 (2 py)	
Years of experience		36 (14)	38 (11)	20	22
Beard	No	18	8		
	Three-day beard	1	3		
	Full beard			1	1

Mean (standard deviation) and number of cases are reported.

*py* pack years.

### Total aerosol emission rates and particle size distribution

**Figure 3:**
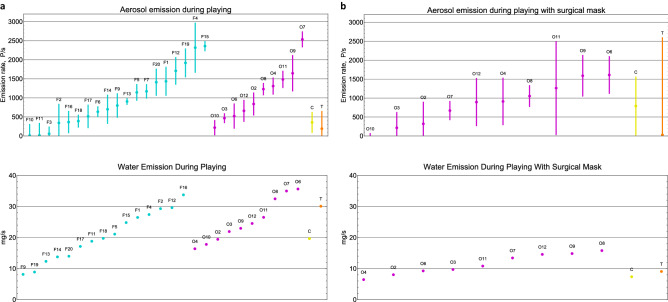
Distribution of aerosol and water emission rates during wind instrument playing without (**a**) and with (**b**) a surgical mask around the bell. *F* flute, *O* oboe, *C* clarinet, *T* trumpet. Numbers indicate participant’s ID. Wind instrument playing lasted 20 min; oboe, trumpet, and clarinet playing with a surgical mask lasted 10 min. Emission rates are reported as particles/second (P/s), water emission rates as mg/s. Error bars indicate standard deviations of estimation uncertainty according to bootstrap calculation.

Aerosol emission rates of a total of 32 musicians playing four different wind instruments covered the range from 11 ± 288 particles/second (P/s) to 2535 ± 195 P/s, average rate ± standard deviation (see Fig. 3a for details). The distribution is uniform for both groups, flute D(19) = 0.28, *p* = 0.08, oboe D(10) = 0.28, *p* = 0.33. Differences between the flute and oboe groups were not detected, U = 81, *p* = 0.51. The aerosol emission rates from playing clarinet and trumpet lay within the lower range of the uniform distributed aerosol emission rates from playing flute and oboe.

**Figure 4:**
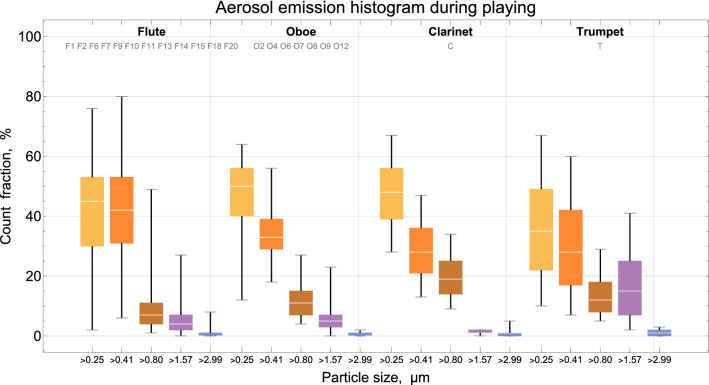
Size distribution of aerosol from playing wind instruments. White split indicates median, whiskers indicate lower and upper fence, box indicates 25% and 75% quantiles.

Analysis of the aerosol particle size distribution (see the histogram in Fig. 4) shows that about 70 ─ 80% of the emitted particles had a size 0.25–0.8 µm. Particles larger than 6.6 µm (droplets) were rarely detected. The seeming differences between the size distributions of aerosols from different instruments are insignificant since the count fraction estimations overlap to great extent. Since particles smaller than 1 µm penetrate deeply into the respiratory tract, down to the alveolar parenchyma, this aspect is of particular importance with respect to the risk assessment of wind instrument playing.

Corporal characteristics like beard wearing had no influence on the aerosol emission rate, probands with such properties did not produce outliers in the distribution.

### Aerosol emission rates of playing wind instruments compared to speaking and breathing

**Figure 5:**
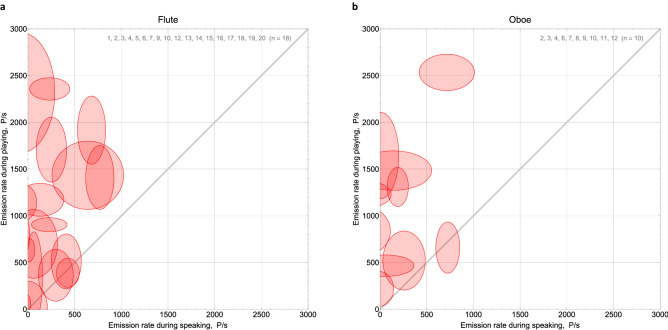
Aerosol emission rates (unit: particles/s) are displayed for the tasks wind instrument playing (**a**) flute, (**b**) oboe, and speaking. The centre point of the ellipse indicates the emission rate from instrument playing on the ordinate and from speaking on the abscissa. The semiaxes of the ellipse indicate the standard deviations of estimation uncertainty for each task, according to bootstrap calculation. The grey lines indicate equal emission rates during both tasks. Ellipses above that line indicate higher emission rates from wind instrument playing than from speaking. The ellipses indicate credibility regions, opacity indicates likelihood.

Aerosol emission rates from music playing were found to be higher than those from speaking and breathing, U = 134, *p* < 0.001 and U = 62, *p* < 0.001 respectively. Interestingly, the sole exception (oboist no. 12) shows similar emission rates for both music playing and speaking (660 ± 274 P/s oboe playing, 725 ± 128 P/s speaking). Figure 5 shows the aerosol emission rates from wind instrument playing vs. from speaking. The ellipses indicate the credibility regions and their opacity indicates likelihood. The vast majority of participants produced higher emissions by playing than by speaking. Aerosol emission rates from speaking varied up to 770 ± 152 P/s and from breathing up to 589 ± 354 P/s. Individual aerosol emission rates from speaking were higher than from breathing in most cases.

### Aerosol protection by using a bell mask

In attempt to reduce aerosol emission, the oboists, the clarinettist, and the trumpeter played with a surgical mask over the bell for 10 min. Due to the shorter measurement time, the relative standard deviations of aerosol emission rates are higher. We find no difference between the aerosol emission rates from playing with and without mask, U = 64, *p* = 0.62. Figure 3b shows a uniform distribution of the aerosol emission rates from playing with a surgical mask around the bell D(12) = 0.17, *p* = 0.84.

**Figure 6:**
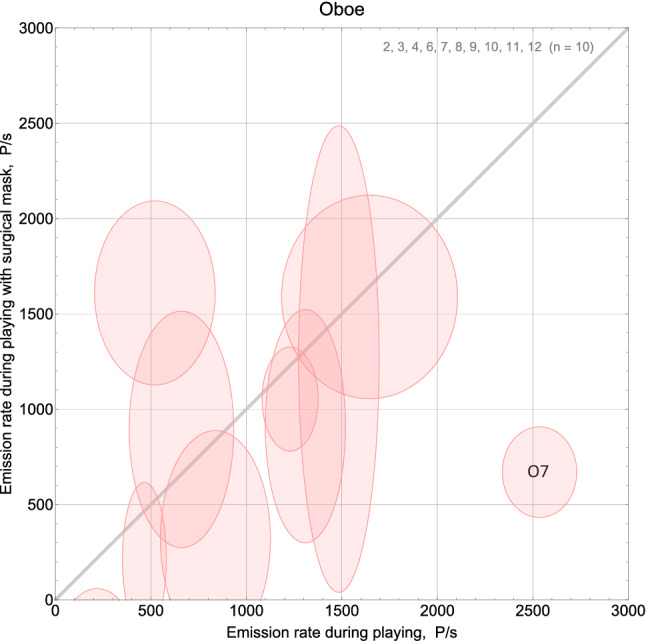
Aerosol emission rates for oboe playing with and without a surgical mask around the bell. Only proband O7 shows essential reduction in aerosol emission when playing with a surgical mask. The grey line indicates equal emission rates during both tasks.

Figure 6 compares the aerosol emission rates from playing with and without mask. The semiaxes of the ellipses indicate the standard deviations of estimation uncertainty. The individual aerosol emission rates are consistent between both tasks (playing with and without mask) as indicated by the ellipses intersecting the gray line in most cases, except one outlier showing the expected reduction of emission by the mask (oboist no. 7).

### Water emission

**Figure 7:**
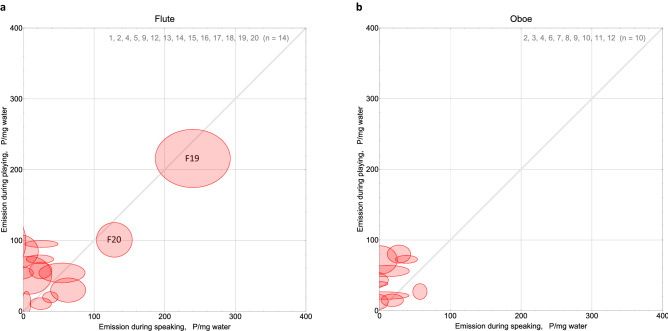
Particle emission per mg water emission during flute (**a**) or oboe (**b**) playing vs. during speaking. Gray lines indicate equal particles-per-water ratios for both tasks. Oboe playing tends to produce larger particles-per-water ratios than speaking while flute playing does not seem to: In diagram (**b**) more ellipses are located above the grey line than on or below it. The ellipses above the line indicate higher ratios from instrument playing than from speaking. In diagram (**a**) the distribution of ellipses with respect to the grey line is almost neutral. F19 and F20 are exceptional dry high emitters.

Complementary to the total aerosol emission rates, we calculated the total water emission rates from the continuous increase of relative humidity during task performance. The water emission rates show a uniform distribution, flute D(15) = 0.15, *p* = 0.83, oboe D(10) = 0.30, *p* = 0.27, like the aerosol emission rates (see Fig. 3). To elucidate the correlation to aerosol emission we calculated their ratio and find that 1 mg emitted water corresponds to ≤ 100 emitted aerosol particles. The ratio was smaller for the task speaking, with two exceptions considered dry high emitters (see Fig. 7).

**Figure 8:**
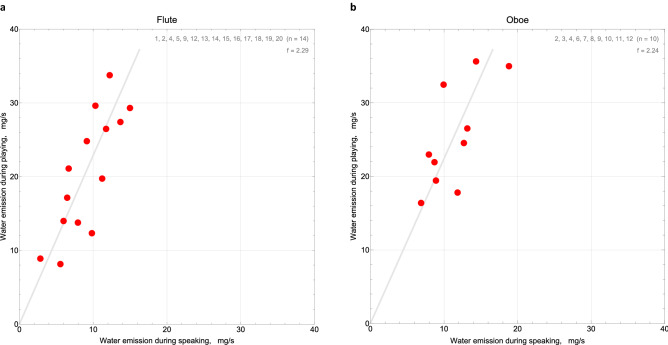
Water emission rates (unit: mg/s) during flute (**a**) and oboe (**b**) playing vs. during speaking. The regression lines have virtual identical slopes of 2.2 for both groups, i.e., water emission generally doubles from speaking to instrument playing.

Since the humidification of exhaled air takes place in the upper respiratory tract we assume that water emission rates provide important information about the respiratory volume and its circulation in airways. We plotted water emission rates during instrument playing versus speaking and found a factor 2.2 between them with high correlation for all wind instruments, flute *r*(12) = 0.80, oboe *r*(8) = 0.71, trumpet and clarinet included (see Fig. 8 and correlation plots in our repository^32^). Water emission universally increased by a factor two from speaking to wind instrument playing, for all probands. The same holds for the comparison of instrument playing to breathing, yielding a universal increase by factor four. If water emission correlates with respiratory volume, the ratios of pulmonary ventilation rates during breathing, speaking, and wind instruments playing appear to be very similar for all participants.

## Discussion

To our knowledge, this study is the first to examine more than two representatives of the same instrument, but a greater and thus more representative number of individuals for two wind instruments (oboe, flute). Moreover, the study design using a hermetically closed probe chamber and standardized playing condition allowed, for the first time, measurement of total aerosol emission rates. Other than in previous studies^26,28^ the different musicians performed the same repertoire piece of music including a variety of dynamics and articulation techniques. Our experiment resembles a real performance with respect to both the scores and the playing time (20 min).

Our results show that typical wind instrument playing generates higher aerosol emissions than typical speaking or calm breathing. During realistic performance, when the musicians play Mozart Concerto as usual, we observe total emission rates in the range previously reported for singing, exceeding 1000 particles per second^33^. This is in line with results by He et al. who found that playing wind instruments in general generates more aerosol than breathing and speaking, whereby the emission rate is dependent on parameters, such as dynamics, articulation, and breathing techniques^26^. Some of the aerosol particle size distributions emitted by our probands during speaking show the laryngeal mode (L-mode) around 2 µm discovered previously^34^ which is not observed in the emissions from instrument playing (s. fig/Histograms/Comparison Laryngeal Mode Histogram.gif in our repository^32^). This is consistent with the fact that playing wind instruments does not involve vocal fold vibrations associated with voicing, thus obviating the physical mechanism underlying the generation of L-mode particles.

The breathing of the musician is the common source of both the loudness of a wind instrument and the aerosol emission. Specifically, exhalation determines sound generation^35–37^ whereas inhalation determines aerosol generation^11^. Higher loudness is produced by greater exhalation flow rate^35,38–40^. A flutist can sustain a note in forte for 8 s without rebreathing, but for 40 s in piano^41^, so the exhalation flow rate increases by an approximate factor five from piano to forte. The aerosol concentration of exhaled air depends on the particle yield of bronchiole fluid film burst which is modulated by the inhalation flow rate^11^. Since aerosol exhalation rate is the product of exhalation air flow rate and particle concentration of the exhaled air, the aerosol emission during playing a woodwind instrument depends on both the inhalation and the exhalation process.

A correlation between loudness and aerosol emission is observed^28^ because the two quantities correlate, each, with exhalation flow rate as modulator. The median aerosol particle number concentrations reported in McCarthy et al.^28^ increase by a factor five from piano to forte, which equals the expected increase of exhalation flow rate during flute playing. The increase of aerosol emission at increasing loudness is, thus, largely explainable by increasing exhalation flow rate. The particles emitted during instrument playing have a similar size distribution as for breathing while speaking and singing would differ thereof by the additional L-mode in the size distribution^11,28,34^.

During musical performance a musician autonomously adapts both inhalation and exhalation to the artistic requirements^42^ so that variation of the inhalation process is ordinary part of wind instrument playing. It introduces an independent modulator of aerosol emission since quicker inhalation, which is typical for flute playing^41^, produces higher aerosol concentrations of the exhalate^11^. Therefore, aerosol emissions from a flute or oboe depend on the playing style in a more complex way than straight correlation with exhalation flow rate or sound pressure. Our probands played a whole Mozart Concerto rather than single notes^28^ and playing all the different phrases with the prescribed dynamics requires most of the instrumental and breathing techniques, whereas sustaining a single tone for 20 s is often feasible without re-breathing.

When comparing the emissions during instrument playing to those from speaking, we refer to the typical loudness associated with playing the Mozart Concerto or reading the Hesse novel aloud, respectively. The music performance was louder than the reading, and the aerosol emission during playing was higher than during reading aloud. It is possible, though, to raise the voice while speaking to produce similar levels of aerosol emission as by playing wind instruments^28^.

The large number of probands playing oboe and flute in our study demonstrated the major individual variability within the two groups. Emission rates show uniform distribution within similar ranges for the two instruments. Unlike previous studies^22,23,26^, no clear allocation of emission rates to the instrument type is possible. We conclude that individual factors dominate the variability of aerosol emission rather than the type of instrument. Outliers from the uniform distribution that might be interpreted as super-spreaders have not been observed, other than in a previous study that detected high aerosol emitting probands during speaking^8^.

In search for individual factors influencing the aerosol emission we found that emission rates do not correlate with body height or weight^32^. Hence, we assume that the breathing technique and the respiratory rate are probably the reason for individual variability of aerosol emission, as outlined in a recent study^43^.

The humidification of exhaled air takes place in the upper respiratory tract^44,45^ whereas aerosol formation is thought to originate deeper in the respiratory tract^11^. Since the humidified air is saturated with water even at high flow rates^46^, water emission likely correlates with pulmonary ventilation rate. Our results indicate higher aerosol-particles-per-water ratios for oboe playing than for speaking. Given that wind instrument playing requires higher pulmonary ventilation rate than speaking, our results are consistent with an increased air exchange in the respiratory tract during wind instrument playing. The required pulmonary volume apparently depends on individual factors, such as vital capacity or breathing technique, which explains the high variability of aerosol emission within the two instrument groups. We found noteworthy correlations between the water emission rates from wind instrument playing, speaking, and breathing indicating that the respiratory volume needed for the respective task might increase similarly for all the different individuals.

Regarding the particle size distribution, most of the particles are < 1 µm in diameter, as found previously for breathing and speaking probands^33^. The SARS-CoV-2 virus has a diameter of 0.13 µm^47,48^. An investigation of the load distribution of SARS-CoV-2 virions in airborne aerosol over different aerosol particle size bins revealed that aerosol particles smaller than 1 µm carried 67% of the total number of genome equivalents per cm^3^ in an air sample^49^. This imposes great risk for long-range COVID-19 transmission since particles < 2 µm reach alveolar parenchyma. Consistently, particles with equilibrium diameters ≤ 1 µm emitted during breathing, speaking, and singing have been causing indoor airborne long-range COVID-19 transmission with attack rates as high as 89% (51 secondary infections among 57 susceptible exposed)^5^. Even particles emitted by infectious individuals during tidal breathing contain aerosolized SARS-CoV-2 RNA copies, 54% of which are contained in fine particles (diameters ≤ 5 μm) labelled here as “aerosol”^50^. Therefore, the “aerosol” particles emitted during instrument playing ought to be considered efficient virus carriers. The emission rates measured here are the most important input parameter of disease transmission risk calculations for the assessment of indoor situations involving the presence of potentially infectious room occupants. Particles with diameters > 6.6 µm were rarely recorded, in agreement with McCarthy et al.^28^, thus being negligible for long-range, airborne disease transmission.

Like other researchers before, we tried to reduce the aerosol emission by masking measures. We masked the bell with a surgical mask on the oboe, clarinet, and trumpet. Except for one oboist, all participants produced similar aerosol emission rates as without mask. A previously described reduction of 50–79%^22,25^ was not observed at the measurement distances used in our study. We assume that aerosol emanated through keyholes and embouchure. Moreover, the most frequent particle class with diameters < 0.8 µm is not filtered efficiently by a surgical mask. Since 4 out of 11 oboists reported a flawed intonation, especially for the notes E5 and F5, while playing with mask we refrain from recommending surgical masks as emission filters for wind instruments.

### Risk assessment of typical woodwind playing situations

Short-range exposure is difficult to model, but easy to mitigate (by social distancing following recommendations, e.g., in Gantner et al. and Hedworth et al.^21,51^). The opposite applies for long-range exposure, in practice. The obvious countermeasures against aerosol transmission are ample fresh air and the wearing of FFP2 masks. However, the efficiency depends strongly on the specific setting. The sole simple rule available is the recommendation to do outdoor whatever can be done outdoor. For indoor occupation, COVID-19 transmission risk can be calculated as described in Reichert et al.^5^ and implemented online for free use: https://hri-pira.github.io^19^.

We apply the framework outlined in Supplement S4 to assess the criticality of a few, typical situations of playing woodwinds. It is assumed that appropriate social distancing excludes short-range exposure so that the infection risk entirely results from long-range exposure. As mentioned before, the hazard in a particular scenario depends on both the individual aerosol emission rate *q* and the infectiousness of the instrument player^52^. In a real situation the disease transmission probability may therefore be a factor 10 less than stated below or even negligible since we assume the worst case of viral load.

For our calculations we assume the maximal infectiousness (${Z}_{50}$ = 833 particles, for the Delta variant) and an aerosol emission rate of *q* = 2500 particles per second while playing, to examine whether *the setting* is safe or not. This question remains important even when an antigen test has been carried out before playing since asymptomatic spreaders may pass at significant rates reported with a sensitivity of 58% to 95%^53^. A safe setting provides the necessary, second line of defense. Vaccination is neglected in the following, thus assuming susceptibility for infection.

#### Lesson at the music school

The teacher and an infectious student have a 60 min lesson in a 200 m^3^ classroom. The student listens 50%, plays 40%, and talks 10% of the time (average aerosol emission rate *q* = 4∙10^6^ /h). Neither wears a mask and the windows remain closed. The resulting long-range infection probability for the susceptible teacher is *p* = 96%. To reduce *p* to 10% by ventilation only, unrealistic 80 air changes per hour (ACH) sustained were necessary. If, instead, the teacher wears a tight FFP2 mask with a filter efficiency of 95% ($\vartheta =0.05$)^54,55^ then *p* = 15%. They may open the door and windows widely for 10 min after half an hour to clear the air from aerosols. Then, *p* = 79% without wearing a mask, or *p* = 7% wearing a mask. To conclude, acceptable safety levels can be reached even at worst-case conditions by

(i) limiting the duration to one hour,
(ii) wearing FFP2 mask whenever suitable, and
(iii) obligatory, thorough airing around half time.

Infection probability with mask is expected around 14% when the space volume of the room is half as large (100 m^3^).

#### Recital

An infectious soloist plays a one-hour program (net playing time) accompanied by two musicians in a 2000 m^3^ ballroom. The audience leaves the room after 90 min, including the encores and applause. Automatic ventilation exhausts air through the ceiling at 2 ACH (4000 m^3^/h) with fresh air streaming inward near the floor. The CO_2_ level stays below 1000 ppm (good air quality) for audiences up to 100 persons. The average aerosol emission rate is *q* = 6∙10^6^/h. The long-range infection risk for susceptible persons is *p* = 3% if they wear FFP2 masks and *p* = 45% otherwise. Given that social distancing prevents accommodation of more than 50 spectators in the ballroom, one secondary infection case is expected when FFP2 masks are worn throughout. The reproductive number in this setting is *R* ≈ 1.

#### Symphonic performance

A woodwind player in an orchestra is infectious. They play symphonic literature, i.e., the duty cycle of woodwinds is average. We assume 30 min net playing time evenly distributed over 90 min concert duration, resulting in an average aerosol emission rate *q* = 3∙10^6^/h. The concert hall has a space volume of 20,000 m^3^. Automatic ventilation exhausts air above stage and auditorium at 2.5 ACH. The long-range infection risk for susceptible persons is *p* = 0.14% when they wear FFP2 masks, and *p* = 3% otherwise, owing to the large air space and fresh air supply.

The basic assumption of full mixing, or perfect aerosol dilution, is questionable in the latter example. Concert stages may have air exhaustion ducts which remove part of the air on stage from the hall before it mixes into the air surrounding the audience. In case of the opposite flow direction, fresh air streaming down from the ceiling, problems may arise, such as local stagnation or recirculation regions with elevated aerosol concentrations^51^. Generally, large premises require consideration of actual flows and should not be assessed using the well-mixed room air assumption. Our example cannot be generalized to other concert halls, based only on their size and total fresh air supply.

## Conclusion

Wind instrument playing, like singing, should be considered more dangerous than speaking or breathing. High interindividual variance of emission rates indicates that physiological factors and playing techniques shape the extent of aerosol formation, rather than the type of instrument. To investigate influencing factors of individual aerosol emission further studies are needed. Our results pave the way to absolute risk calculations for oboe and flute playing in typical situations.

## Supplementary Information

Supplementary Information.

## Data Availability

Data sets, supplementary data and figures are available in our repository^32^, https://doi.org/10.5281/zenodo.6323568.
