# Supplementary material for: Aerosol emission from playing wind instruments and related COVID-19 infection risk during music performance
Source: Sci Rep. 2022 May 21;12:8598. doi: 10.1038/s41598-022-12529-2 (PMC9124212; doi:10.1038/s41598-022-12529-2)
Supplement: Supplementary file 1 — Supplementary Information. [file 41598_2022_12529_MOESM1_ESM.pdf]

Supplementary Material to

Aerosol emission from playing wind instruments and related COVID-19 infection risk during music performance

Firle, Carl+ * [1 A]; Steinmetz, Anke+ [2 B]; Stier, Oliver+ [3 C]; Stengel, Dirk [4, 6 D]; Ekkernkamp, Axel [2, 5, 6 E]

+contributed equally
*corresponding author (c.firle@gmx.eu), 0000-0003-3233-178X

1. GP practice, Dr. Claudia-Isabella Wildfeuer, 10715 Berlin, Germany
2. Physical and Rehabilitation Medicine, Dept. of Trauma, Reconstructive Surgery and Rehabilitation Medicine, University Medicine Greifswald, Greifswald, Germany
3. Siemens AG, Technology, 13623 Berlin, Germany
4. BG Kliniken – Klinikverbund der gesetzlichen Unfallversicherung gGmbH, Berlin, Germany
5. Dept. of Trauma, Reconstructive Surgery, and Rehabilitation Medicine, University Medicine Greifswald, Greifswald, Germany
6. BG Klinikum Unfallkrankenhaus Berlin gGmbH, Berlin, Germany

A. corresponding author: c.firle@gmx.eu, 0000-0003-3233-178X
B. anke.steinmetz@med.uni-greifswald.de, 0000-0002-7118-3372
C. oliver.stier@siemens.com
D. dirk.stengel@bg-kliniken.de
E. axel.ekkernkamp@med.uni-greifswald.de, axel.ekkernkamp@ukb.de

Supplement 1

Device specifications and operation

The Grimm *Dust-Decoder 11-D* and *Portable Aerosol Spectrometer Model 1.109* measure at 1.2 l/min sampling flow rate and count particles during periods (measurement cycles) of 6 s duration, i.e., within air samples of 120 ml volume, by laser light scattering. During each measurement cycle the laser power changes (multiplex mode) to measure
particle sizes 0.25 – 2.5 µm at high power during 1 s (20 ml sampling volume) and
particle sizes 2.5 – 32 µm at low power during 5 s (100 ml sampling volume).
The optical particle counter detects single particles so that the concentration resolution is 1 particle (P) per 20 ml (= 50 P/l) for particle sizes 0.25 - 2.5 µm and 1 particle per 100 ml (= 10 P/l) for particle sizes 2.5 – 32 µm.

During one measurement cycle in the multiplex mode two integer numbers of particles are obtained, n_1 and n_2, the one counting particles ≤ 2.5 µm contained in 20 ml air (n_1) and the other counting particles ≥ 2.5 µm contained in 100 ml air (n_2). Categorization of the detected particles by their size into a total of 31 size bins yields partitions of n_1 and n_2 which are continuously streamed by the spectrometers as 31 size channels every 6 s. The Grimm software returns the counting results for all particle size bins in the unit P/l so that all concentration values are integer multiples of 50 or 10 P/l, respectively.

An intrinsic property of the finite volume sampling method is random fluctuation of all count numbers. The probability that a particle is contained in the air portion analyzed by the spectrometer (sampling volume V_s) is $p = V_s/V$ with V being the probe chamber space volume. In our measurements, $p = 1 \cdot 10^{-6}$ ($5 \cdot 10^{-6}$) for particles ≤ 2.5 µm (≥ 2.5 µm). Particle counts are distributed as binomial(N, p) where $N = c\,V$ is the total number of particles in the probe chamber air and c is their average concentration. For such large numbers N (≥ 10^6) and small values of p the binomial distribution is well approximated by the Poisson distribution with mean $\mu = Np = c\,V_s$.

Hence, the measured values n_1 and n_2 are independent random draws from two different Poisson distributions [16] whose expectation values, μ_1 and μ_2, are proportional to the air concentrations of particles ≤ 2.5 µm (≥ 2.5 µm) by the factors 50 P/l (10 P/l), respectively: $c_i = \mu_i/V_s$. This conversion is performed by the Grimm software.

The point estimation of the Poisson mean is $\mu_i = n_i$ and a useful confidence interval (CI) [56] is

$$\mu_i \ \epsilon \ \left[\, \max(0,\, n_i - \sqrt{n_i + 1}) \ ; \ n_i + \sqrt{n_i + 1}\, \right] \qquad \text{(Eq 1)}$$

which contains the true value of μ_i with a coverage probability of at least 60%. For $\mu_i < 1$ the coverage probability is ≥ 92%. At low particle concentrations, $c > 0$, μ becomes so small that $n_i = 0$ particles are detected accidentally, which does not imply the concentration is zero, though. In this case, Eq (1) yields the CI $[0;\ 1/V_s]$ for the particle air concentration c. At increasing count values n_i the CI coverage probability approaches 68.3% so that the (symmetric) CI is suited as surrogate ±1σ error bar of the count number (σ is the standard deviation). The associated signal-to-noise ratio (SNR) is

$$\mathrm{SNR} = \frac{n_i}{\sqrt{n_i + 1}} \qquad \text{(Eq 2)}$$

Hence, count values $n_i < 5$ carry little information about the particle concentration.

The Grimm Aerosol Spectrometer 1.109 partitions n_1 by subdividing the particles into the size bins (lower bounds, µm) 0.25, 0.28, 0.3, 0.35, 0.4, 0.45, 0.5, 0.58, 0.65, 0.7, 0.8, 1.0, 1.3, 1.6, 2.0, 2.5 and n_2 by subdivision into the bins 2.5, 3.0, 3.5, 4.0, 5.0, 6.5, 7.5, 8.5, 10.0, 12.5, 15.0, 17.5, 20.0, 25.0, 30.0, 32.0 whereas device model 11-D partitions n_1 by subdividing the particles into the size bins 0.253, 0.298, 0.352, 0.414, 0.488, 0.576, 0.679, 0.8, 0.943, 1.112, 1.31, 1.545, 1.821, 2.146, 2.53 and n_2 by subdivision into the bins 2.53, 2.982, 3.515, 4.144, 4.884, 5.757, 6.787, 8.0, 9.43, 11.12, 13.1, 15.45, 18.21, 21.46, 25.3, 29.82, 35.15. The bin 2.5(3) is measured at both laser powers and averaged.

Given the low particle concentrations c prevailing in our measurements the high size resolution is not advantageous. Since n_1 and n_2 are small, zero counts occur in abundance with such narrow size bins. Therefore, we chose to merge adjacent bins a posteriori to obtain a coarser subdivision which, in addition, is consistent between the two spectrometer models. When size bins pertaining to the same air sample merge, their counts n_i add and Eqs (1) and (2) apply to the total. When two bins pertaining to different air samples merge their count values n_i are incompatible, as they refer to different sampling volumes V_s. In this case the concentration values c_i are added, taking their error bars due to Eq. (1) into account. The standard deviation of c_i is approximated by $\sqrt{n_i + 1}/V_s$ and the variances add since the c_i are statistical independent.

The new, merged size bins are 0.25, 0.41, 0.80, 1.57, 2.99, 6.64, 12.80, 25.15, 29.91, as well as one called “aerosol” (all particles < 6.64 µm) and another called “droplets” (all particles ≥ 6.64 µm). The counts in the “droplets” bin were mostly zero, thus we focus on the “aerosol” bin. Except for particle size histograms, we report and discuss concentration measurements referring to the “aerosol” bin only, i.e., concerning the airborne particles < 6.64 µm. The latter size bound differs from the 2.5 µm most used in the literature. Since particles in the size range 2.5 — 6.64 µm were detected most rarely in our measurements, this inconsistency between our definition and the common one is negligible.

The thermo-hygrometer *Voltcraft DL-220THP* has a resolution of temperature of 0.1 °C, of relative humidity 0.1%, and of air pressure 1 hPa. The measuring range of temperature is -30 to 60 °C, of relative humidity 0 to 100%, of air pressure 300 to 1200 hPa. Accuracy of temperature is 0.5 °C, of relative humidity 3.5%, and of air pressure 2 hPa. The total amount of water solved in the chamber air as steam is calculated, in g, from the temperature θ (°C), the relative humidity x (%), and V (m³) by

$$m_{\text{water}} = \frac{2.16675\, p_s(\theta)}{273.15 + \theta}\, V\, x$$

where $p_s(\theta)$ is the saturation vapor pressure of water, in hPa [57].

The anemometer *testo 405 i* senses air flow with a resolution of 0.01 m/sec. The measuring range is 0 to 30 m/sec, the accuracy is ± 0.1 m/sec + 5% of the mean for velocities between 0 and 2 m/sec and ± 0.3 m/sec + 5% of the mean for velocities of 2 to 15 m/sec.

The microphone used was *TLM 102 bk* produced by Neumann. It was linked to the Zoom recorder H4n Pro. Output was saved as mp3-file with a bit rate of 128 kbps.

Supplement 2

Measured quantity

Prerequisite for correct measurement of total emission is that the quantity of aerosol does not change between origin and detection, i.e., along the path from the proband's respiratory tract to the spectrometer. Once formed, exhaled aerosol particles undergo significant changes of their water content by competing processes of hygroscopic growth and desiccation [15]. It takes in the order of a second for an airborne particle to attain a stable equilibrium diameter. Any earlier measurement of particle sizes or masses would yield nonstationary, irreproducible quantities while later sampling would map the equilibrium state, rather than the original one.

In contrast, the number of particles is largely preserved from formation to detection, thus being the more reliable measure for amounts of exhaled aerosols. A minor correction is due to surface deposition of particles, as outlined in Supplement 3. The spectrometers we used return both mass and number distributions. We consider the latter since mass values in most cases require model-based restoration of the original particle size which would introduce speculation to the measurement result. The size distributions we obtained have undergone an unknown, but continuous, transformation and depend on the relative humidity inside the probe chamber. Most droplets larger than 30 µm in diameter have got lost due to gravitational settling. The size distributions of smaller, airborne particles are similar to other measurements at the same relative humidity.

Measurement accuracy

The SNR of particle counting measurements is intrinsically limited by random count fluctuations resulting from finite volume sampling. According to Eq (1) detection of n particles smaller than 2.5 µm indicates an air concentration of $50\left(n \pm \sqrt{n+1}\right)$ P/l whereas n particles larger than 2.5 µm indicate a concentration of $10\left(n \pm \sqrt{n+1}\right)$ P/l. For example, a concentration of 150 P/l for diameters 0.4 – 0.8 µm is measured with a 1σ uncertainty of ± 100 P/l since n = 3. Sufficient precise values are obtained by averaging dozens of such single values. This entails long measurement times and leads to the chosen performance duration of 20 min per task.

During this period each of the three spectrometers measures 204 count values per size bin, in time intervals of 6 s, as shown in Fig. S1. These are averaged in groups of 34 subsequent values to obtain six new data points equidistant in time, which are shown as gray symbols in Fig. S2. They represent the average particle concentrations during periods of 200 s, each, which is the time scale for uniform distribution of airborne aerosol particles inside the probe chamber.

By assumption of well mixed air and aerosols, particle counts measured simultaneously at the three spectrometers represent the same, global particle concentration at that time. Thus 3·34 = 102 original count values merge to one concentration point estimate whose uncertainty is Gaussian (meeting the Lindeberg condition of the central limit theorem) and reduced by the factor 10 as compared to single count values (by Eq 2). These estimates are shown in Fig. S2 as blue symbols.

The limit of detection (LOD) is the smallest air concentration of particles that is safely distinguishable from zero. The LOD for particle sizes < 2.5 µm is 5 P/l and the LOD for particle sizes ≥ 2.5 µm is 1 P/l. At these concentrations the Poisson mean is µ = 0.10 and the probability is 0.00004 to accidentally detect a total of 0 particles in 34 subsequent measurements on each of the three spectrometers, i.e., to obtain 102 zero counts.

A straight line representing the expected linear increase (see Supplement 3) of particle air concentration over time is fitted to the six consolidated data points shown as blue symbols in Fig. S2. The least-squares fit takes the individual error bar magnitudes (uncertainties) into account and is shown as red line. Its slope, multiplied by the chamber space volume, is the point estimate of the average particle emission rate of the proband, as outlined in Supplement 3. The emission rate uncertainty is calculated using the bootstrap method [58] and reported as standard deviation of the marginal distribution of the emission rate, with the initial concentration c_0 as nuisance parameter. The 68.3%, 90%, and 95% credibility regions of the fit are shown in Fig. S2 as light-red shaded regions.

By the procedure described here, 612 single particle counts referring to one particle size bin have been combined to one emission rate value and its $\pm 1\sigma$ error bar. The procedure is applied to each size bin separately to calculate the dependence of emission rate on the particle size.

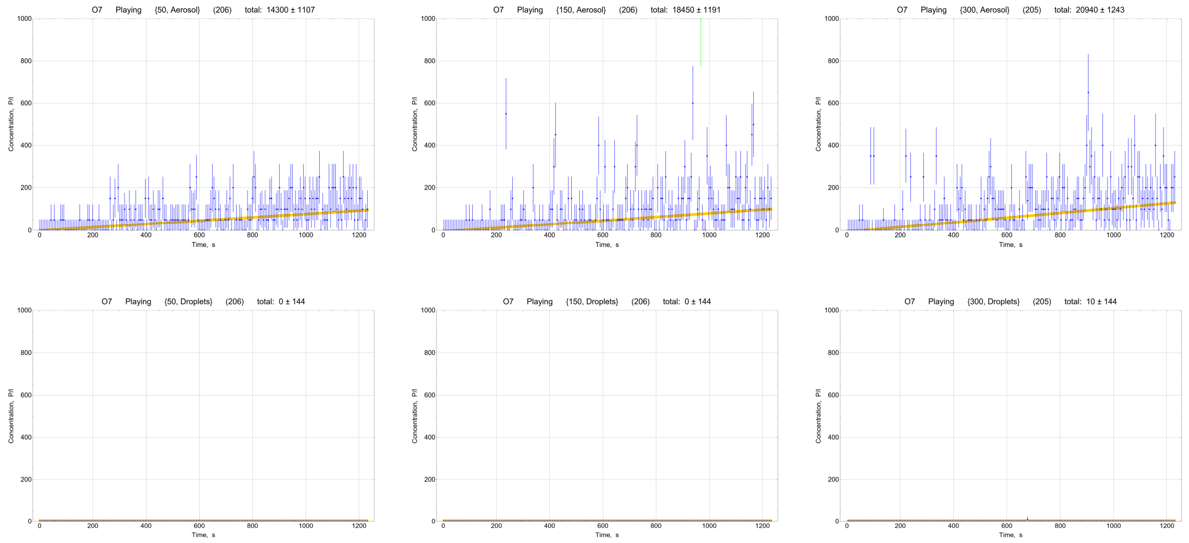

Supplementary Figure S1. Single measurement values for “aerosol” (≤ 6.6 µm, top row) and “droplet” particles (> 6.6 µm, bottom row), measured by the three spectrometers at 50, 150, and 300 cm, respectively (from left to right). The concentration point estimates are indicated by blue dots, the ±1σ error bars according to Eq (1) are shown as blue vertical lines. Green symbols are considered outliers, the associated values have been ignored in further computation. Orange lines show the trend as guide to the eye. The graphics visualize the typical SNR of our measurements. Also typical is the sparsity of particles larger than 6.6 µm shown in the three plots in the bottom row.

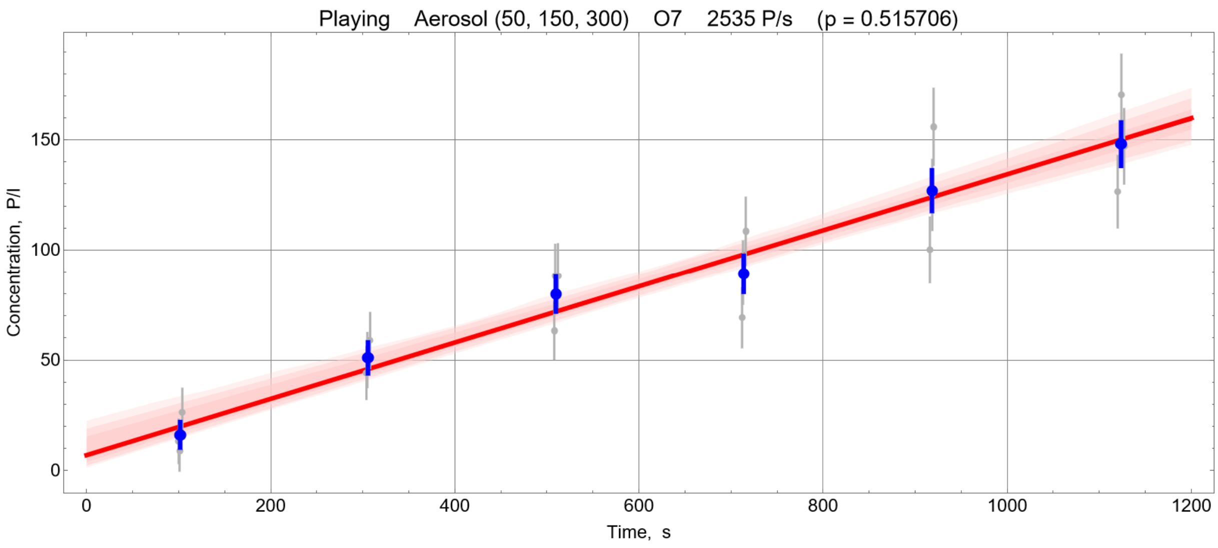

Supplementary Figure S2. Time-averaged "aerosol” concentrations at the three spectrometers (gray) and position-averaged concentrations (blue) obtained by averaging simultaneous results from the spectrometers. The red line is a straight-line least-squares fit, the significance level according to the χ^2-test is reported in the plot label. Shaded regions indicate the credibility regions of the straight line for coverage probabilities 68.3%, 90%, and 95%, respectively. They visualize the uncertainty of the slope according to bootstrap estimation. The standard deviation of this uncertainty, multiplied by the probe chamber volume, is reported as ±1σ error bar of the aerosol emission rate.

Supplement 3

Estimation of aerosol emission rates from concentration measurements

The experimental setup shown in Fig. 1 has been designed to isolate and collect respiratory aerosols, i.e., to exclude other sources of aerosol or fine-dust and to prevent loss of the exhaled aerosols. Before each measurement the air inside the probe chamber was purged with prefiltered room air and additionally purified using the stand-alone air cleaner inside, until the aerosol concentration fell below 50 P/l on all three spectrometers. The special clothing used warrants low air contamination with non-respiratory particles while the foil tent protects the measurement volume from intrusion of foreign particles.

The number concentrations of particles were below 1 P/cm³ at all times so that particle coagulation is insignificant [59]. Exhaled aerosol particles do not evaporate completely since they attain equilibrium diameters. Hence, their number decreases only upon removal from the measurement volume, i.e., when particles pass the boundary surface surrounding the latter.

If the probe chamber had an opening, airborne particles passed the imaginary boundary and wafted outside driven by outward air motion, in exchange for fresh air flowing inwards. The resulting particle loss depends on the air change rate λ between the chamber volume and the exterior, such that λ is the particle number loss coefficient due to ventilation.

Replacement of the hypothetical opening by the actual wall leads to suppression of the net air flow perpendicular to the volume boundary, while preserving the sink property of the boundary surface: Particles reaching the wall by accidental impingement are deposited due to adhesive forces [16]. The wall boundary layer is depleted of particles and creates a local concentration gradient causing a net particle diffusion toward the wall. The wall impingement rate of airborne particles is proportional to their air concentration, hence, losses due to wall deposition are accounted for by a particle decay rate loss coefficient β having the same unit and effect as λ. Larger droplets are efficiently removed from the chamber volume by deposition on the floor following gravitational sedimentation. Therefore, particles larger than 6.6 µm in diameter were rarely detected in our measurements.

The particle concentrations measured at different spectrometer positions are largely consistent, as seen from Fig. 2 and the additional data plots provided in our repository [32]. The apparent absence of systematic position dependence is interpreted as sufficient mixing of aerosols with the chamber air. We treat aerosol as instantly reaching uniform concentrations depending only on time, idealizing the high drift velocity, reach, and stability of exhaled, airborne aerosol particles described by Chong et al. [17]. The air concentration of particles emitted by the proband, $c(\tau)$, evolves according to

$$\frac{\mathrm{d}}{\mathrm{d}\tau}c(\tau) = \frac{Q}{V} - (\lambda + \beta)\, c(\tau) \qquad \text{(Eq 3)}$$

from the initial concentration $c(0) = c_0$ (unit: particles per m³), τ is the time variable (unit: h). λ and β have the unit h^{-1} and V is the space volume of the probe chamber in m³. Q is the wanted aerosol emission rate in particles per hour. Eq (3) suggests that Q be derived from the slope s of the curve $c(\tau)$ during the measurement period $\tau = 0 ... t$ with $t = 0.333$ hours.

The probe chamber is sealed after the proband has entered, so that $\lambda = 0$ during the measurements. For any given particle concentration c_0 at the beginning of the measurement $c(\tau)$ changes monotonously, approaching the stationary concentration

$$c(\infty) = \frac{Q}{\beta V} \quad \text{(Eq 4)}$$

The average slope of $c(\tau)$, i.e., the temporal expectation value of $\mathrm{d}c(\tau)/\mathrm{d}\tau$ during measurement is

$$s = \frac{Q}{V} \frac{1 - \mathrm{e}^{-\beta t}}{\beta t} \left[1 - \frac{c_o}{c(\infty)}\right] \quad \text{(Eq 5)}$$

from solution of Eq (3). So, if $c_0 < c(\infty)$ the concentration increases, and if $c_0 > c(\infty)$ it decreases. In absence of wall deposition losses $\beta = 0$ and the curve $c(\tau)$ is a straight line with the slope $s = Q/V$. For $\beta > 0$ the curve $c(\tau)$ has a curvature revealing permanent fading of particles.

The low SNR of $c(\tau)$ in our measurements prevents distinction between a straight and a curved line. A possibility to estimate β from our data is analysis of measurements where the particle concentration appears to remain unaltered during the recording period. Then, $c_0 = c(\infty)$ so that

$$\beta = \frac{1}{V} \frac{Q}{c_0} \quad \text{(Eq 6)}$$

We observed constant particle concentration while probands were calmly breathing through the nose. The respective measurement curves $c(\tau)$ are shown in Fig. S3. Particle emission rates of healthy probands breathing through the nose have been published previously [5] and found to have a lognormal distribution. Based on those data we assume that our probands emitted approx. 87.000 particles per hour, as median value.

With the median values for c_0 and Q, Eq (6) yields the median estimate β = 0.12 h^{-1}. This value is plausible in view of observations that $PM_{2.5}$ combustion smoke particles in a furnished room deposit at 0.10 h^{-1} [60]. Our estimate of β is also consistent with particle deposition measurements on smooth aluminum and wallpaper surfaces in still air [61] and on gypsum board [62].

According to Eq (4) stationary particle concentrations exceed the initial concentration, $c_0 < c(\infty)$, whenever $Q \geq 25$ P/s. Hence, the measurement curves $c(\tau)$ have positive slope s for all tasks, except breathing. Straight line fits to measured data may accidentally produce negative slope values s because of local air motion or the intrinsic counting noise. True negative slopes of c(τ) are only possible at emission rates below 25 P/s, though, which produce respiratory particle concentrations too low to distinguish them from background signal. Therefore, negative fit curve slopes are not physical plausible and need to be corrected to zero. Accordingly, the emission rate display range is clipped at zero in all plots.

The curvature of $c(\tau)$ due to wall deposition loss reduces the average slope s compared to the case of no particle loss. The relative decrease $s/(Q/V)$ is calculated from Eqs (4, 5) and depends on the particle emission rate Q, as displayed in Fig. S4. For emission rates $Q >$ 180 (400) P/s the effect is minor since the slope is at least 80% (90%) that without particle loss. Hence, the approximation $Q \approx s\,V$ is sufficient accurate an estimate of the aerosol emission rate in our measurements, it underestimates Q by 25%, at most.

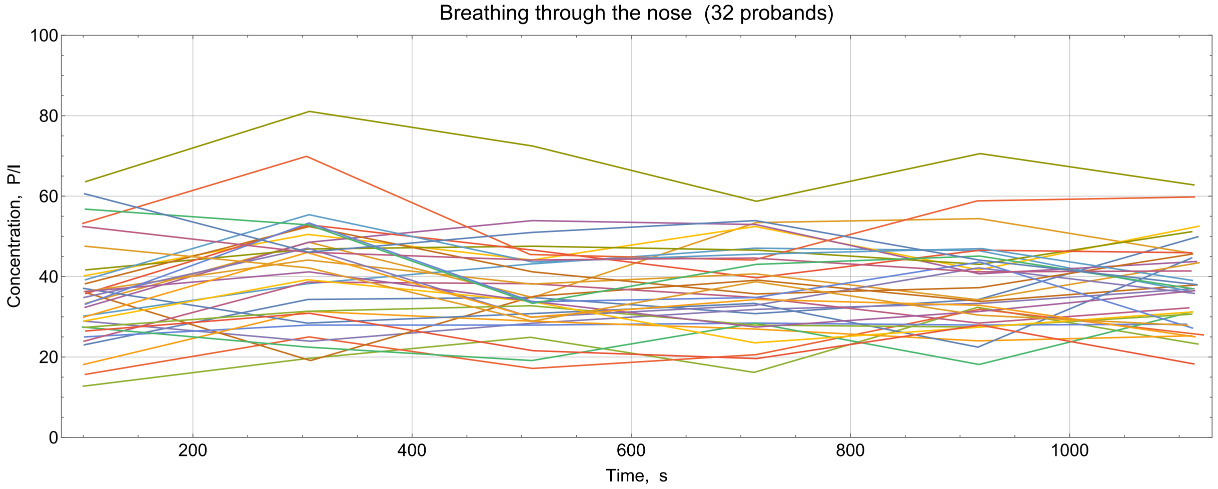

Supplementary Figure S3. Particle concentrations produced during calm breathing. The individual measurements $\boldsymbol{c(\tau)}$ are, each, represented by the six time- and position-averaged data points (see Figure S2) connected by straight lines as guide to the eye. Different line colors distinguish between the probands. All curves pass the χ²-test (p = .05) of the hypothesis that deviations from $\boldsymbol{c_0 = c(\infty)}$ are random. The average over the probands is $\boldsymbol{c_0} =$ 37.700 P/m³, the distribution is $\boldsymbol{c_0 \sim \mathrm{lognormal}(10.5, 0.3)}$ P/m³.

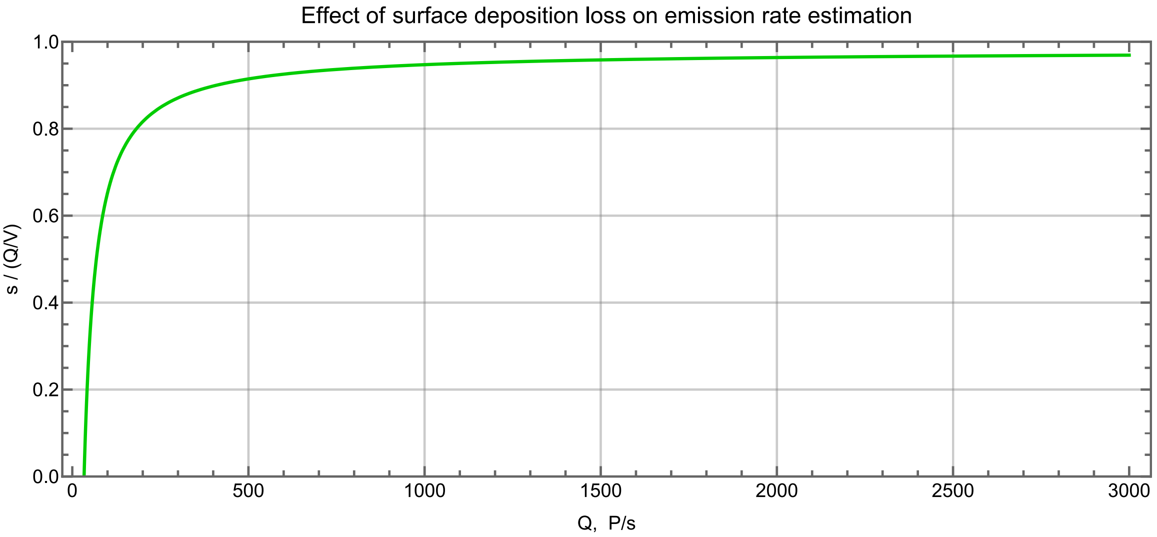

Supplementary Figure S4. Ratio of the average slopes of measurement curves $c(\tau)$ with and without particle loss due to wall deposition, calculated from Eq (5) for $c_0 = 50$ P/l, $t = 20$ min, $V = 19.9$ m³, and β = 0.12.

Supplement 4

Calculation of transmission risk

The exponential dose-response model relates the probability p of infection to the intake dose of pathogens, Z, by

$$p \;=\; 1 - \mathrm{e}^{-Z/\gamma} \;=\; 1 - 2^{-Z/Z_{50}} \qquad \text{(Eq 7)}$$

where γ is a disease-specific, characteristic dose referred to as 'quantum of infection' [63] and $Z_{50} = \gamma \ln 2$. The common unit of Z, Z_{50}, and γ is free to choose; it could be, for instance, RNA copies, plaque-forming units, or aerosol particles. Z_{50} is neither a threshold dose, nor a tolerance dose, but determines how fast infection risk statistically increases at increasing inhalation dose Z. Z_{50} quantifies the average susceptibility of non-vaccinated individuals to infection by the respective virus strain. If the pathogen concentration of the inhaled air is constant over time, Eq (7) can be written as the Wells–Riley equation [18].

Recently, Z_{50} has been estimated for SARS-CoV-2 as number of (partly) virus-laden aerosol particles whose equilibrium diameters are in the range 0.3 µm to 5 µm [5], the same size range as in our present work. This makes it possible to use the aerosol emission rates determined here for calculation of absolute COVID-19 transmission probabilities in situations typical for woodwind playing: A lesson at the music school, a recital, and a symphonic performance in a concert hall. These scenarios involve non-stationary accumulation of aerosols in the air which is not described by the Wells–Riley equation.

For the SARS-CoV-2 lineage B.1.1.7 (Alpha) Z_{50} = 1166 (median, 95% CI: 700 – 1942) aerosol particles have been proposed as likely lower bound for the characteristic dose of infection [5]. The lineage B.1.617.2 (Delta) has a factor 1.4 – 1.6 higher transmissibility than Alpha [64–66]. This factor is composite of a higher infectiousness (expressed by Z_{50}) and a longer duration of infectiousness (resulting in more opportunities for secondary infections). We assume an increase of infectiousness over the Alpha variant by factor 1.4 and apply the consideration of Reichert et al. to derive Z_{50} = 833 aerosol particles with equilibrium diameters 0.3 µm – 5 µm for the Delta variant [5].

The infectious power of a given number Z of aerosol particles depends on the number of active virus contained. For SARS-CoV-2, highest infectiousness has been shown between two days before and the day of symptom onset [67–70]. The virion concentration varies by one or two orders of magnitude, depending on the individual and the infection stadium [71,72].

Accordingly, transmission risk calculations based on the above Z_{50} value apply to the worst case that the woodwind player performs during the phase of maximal infectiousness. Few days earlier, or a week later, the total viral load of aerosol particles released could be a factor 10 to 100 smaller. Hence, our calculations only indicate how dangerous instrumental play could be in the extreme case. They are apt to assess the criticality of environments and the efficacy of safety measures, whereas they cannot specifically predict case numbers of secondary infection in the context of music performances.

The inhalation dose Z in Eq (7) is the number of (partially) infectious aerosol particles inhaled by a susceptible person during the period of exposure. It is calculated assuming instant, perfect mixing between aerosol and room air as outlined in Reichert et al. [5]. Z may be scaled by the inward transmissivity of a face mask worn which reduces infection probability by the factor ϑ = 1 – (filter efficiency). When no mask is used, $\vartheta = 1$, and Z remains unaltered.

Influencing factors of the long-range infection risk p are the use of FFP2 masks for self-protection (ϑ) and the

1. total emission rate of infectious aerosol particles, q
2. exposure duration
3. room space volume
4. ventilation or airing.

In our study we quantify the first factor. To observe the scatter between individuals we measured q for 19 flute and 11 oboe players performing the same task under identical conditions. The second to fourth factor depend on the scenario, as outlined in the discussion chapter.
